# Biophysical characterization of light-gated ion channels using planar automated patch clamp

**DOI:** 10.3389/fnmol.2022.976910

**Published:** 2022-08-09

**Authors:** Elena G. Govorunova, Oleg A. Sineshchekov, Leonid S. Brown, John L. Spudich

**Affiliations:** ^1^Department of Biochemistry and Molecular Biology, Center for Membrane Biology, The University of Texas Health Science Center at Houston McGovern Medical School, Houston, TX, United States; ^2^Biophysics Interdepartmental Group, Department of Physics, University of Guelph, Guelph, ON, Canada

**Keywords:** channelrhodopsins, optogenetics, potassium channels, kalium channelrhodopsins, automated patch clamp, light-gated channels

## Abstract

Channelrhodopsins (ChRs) are proteins that guide phototaxis in protists and exhibit light-gated channel conductance when their genes are heterologously expressed in mammalian cells. ChRs are widely used as molecular tools to control neurons and cardiomyocytes with light (optogenetics). Cation- and anion-selective ChRs (CCRs and ACRs, respectively) enable stimulation and inhibition of neuronal activity by depolarization and hyperpolarization of the membrane, respectively. More than 400 natural ChR variants have been identified so far, and high-throughput polynucleotide sequencing projects add many more each year. However, electrophysiological characterization of new ChRs lags behind because it is mostly done by time-consuming manual patch clamp (MPC). Here we report using a high-throughput automated patch clamp (APC) platform, SyncroPatch 384i from Nanion Technologies, for ChR research. We find that this instrument can be used for determination of the light intensity dependence and current-voltage relationships in ChRs and discuss its advantages and limitations.

## Introduction

For control of neural circuitry, light provides a much higher spatial and temporal resolution than conventional pharmacological interventions. Microbial rhodopsins are photoactive proteins, in which photon absorption triggers diverse molecular functions, from protein-protein interaction to ion transport (Govorunova et al., 2017; Rozenberg et al., 2021). Expression of microbial rhodopsin genes in animal and human cells enables photocontrol of their biochemical and physiological properties, which is now referred to as optogenetics (Deisseroth, 2015). So far, the most impactful optogenetic application has been manipulation of the electrical activity of neurons and cardiomyocytes using channelrhodopsins (ChRs), photoactivation of which results in passive transmembrane conductance. ChRs are widely used in neuroscience and cardiology research, and their potential to treat Alzheimer’s disease (Cui et al., 2020), Parkinson’s disease (Yu et al., 2020), epilepsy (Paschen et al., 2020), cardiac arrythmias (Cheng et al., 2020), and many other neurological, psychiatric and cardiological disorders has been demonstrated in animal models. A report of successful partial vision restoration in a blind human patient using a ChR was published last year (Sahel et al., 2021).

Different optogenetic applications require ChR variants with different properties, most important of which is ion selectivity. ChRs that generate H^+^ and Na^+^ influx in mammalian cells (cation-conducting ChRs, or CCRs) have been found as phototaxis receptors in chlorophyte algae (Nagel et al., 2002, 2003; Sineshchekov et al., 2002) and are used to stimulate neuronal spiking with light (Boyden et al., 2005; Deisseroth, 2011). ChR2 from the green alga *Chlamydomonas reinhardtii* (*Cr*ChR2) and its mutants are the most frequently used tools for this purpose (Wietek and Prigge, 2016). Anion-conducting ChRs (ACRs), found in cryptophyte algae and some other marine protists (Govorunova et al., 2015, 2020; Oppermann et al., 2019; Rozenberg et al., 2020), are used to inhibit neuronal activity. Very recently, KCRs, a new family of ChRs with a high selectivity for K^+^ over Na^+^ has been discovered and used to silence neurons with light (Govorunova et al., 2022). ChRs with red-shifted absorption spectra are generally preferable for optogenetics (Klapoetke et al., 2014; Govorunova et al., 2020), because red light more deeply penetrates biological tissue. However, use of ChRs in combination with fluorescent voltage sensors for all-optical electrophysiology creates a demand also for blue light-absorbing ChRs (Fan et al., 2020). Finally, precise optical control of neurons firing at high frequencies (Keppeler et al., 2018) and prolonged inhibition of neuronal activity (Stahlberg et al., 2019) need ChRs with fast or slow photocurrent kinetics, respectively. These requirements call for novel ChRs variants with the desired properties either identified in nature or engineered in the laboratory.

Several hundred natural ChRs variants have been identified (Rozenberg et al., 2020; Govorunova et al., 2021), and many more are reported each year in ongoing genome/transcriptome sequencing projects (Bork et al., 2015). Conventional manual patch clamp (MPC), generally used for electrophysiological characterization of ChRs, requires considerable expertise and is time-consuming. Recently, high-throughput, automated patch clamp (APC) platforms have become increasingly popular in ion channel research and drug discovery (Bell and Fermini, 2021; Obergrussberger et al., 2021). The SyncroPatch 384i platform from Nanion Technologies uses a “bottom-up” planar configuration to allow compatibility with a multiwell plate format to enable simultaneous recording from 384 wells (in a one-module configuration) or 768 wells (in a two-module configuration). Each well of a multiwell plate has one or several small apertures (holes) in the bottom surface made of borosilicate glass. A suspension of target cells is introduced into the wells, after which a negative pressure is applied to capture the cells in the apertures and form Gigaohm (GΩ) seals. Integrated robotic operation for handling of cells, solutions, and compounds provides standardization of all procedures. The SyncroPatch 384i offers unprecedented high throughput close to that of non-electrophysiological screening techniques such as ion-flux measurements and fluorescence assays, with the added benefit of real-time monitoring of channel activity. This instrument and its earlier modifications have already been used to probe voltage- and ligand-gated ion channels in a variety of cell types (Brinkwirth et al., 2020; Potet et al., 2020; Obergrussberger et al., 2022).

We were granted access by Nanion to a SyncroPatch 384 for a 9-months pilot program to evaluate its utility for ChR research. We used this APC platform to characterize photocurrents from three ChR variants. *Hc*KCR1 from *Hyphochytrium catenoides* is a member of a recently discovered class of ChRs that exhibit higher relative permeability to K^+^ than to Na^+^ detected by MPC (Govorunova et al., 2022). *Hc*CCR is a previously uncharacterized paralog from the same organism, and *Cr*ChR2, the most-studied CCR, was included for comparison. Here we report the results obtained with these three ChRs and discuss the advantages and disadvantages of the SyncroPatch system for ChR research.

## Materials and methods

The genes encoding the three *H. catenoides* rhodopsins were identified by Leonard et al. (2018), and refinement of the sequences of *Hc*KCR1 and *Hc*KCR2 has been described by us recently (Govorunova et al., 2022). Initially, we obtained the predicted protein sequence for *Hc*CCR from the database provided by Leonard and Richards,^1^ the file hyphochytrium_catenoides_predicted_proteins_renamed _modified. Upon close inspection of an alignment of the predicted transmembrane (TM) domains of these rhodopsins it became clear that the *Hc*CCR sequence is missing part of helix 5. This prompted us to reconfirm the sequence of its TM domain by performing a TBLASTN search of the whole-genome sequencing (WGS) data for *H. catenoides* (Accession FLMG00000000.1 and CAFC00000000.2) at the National Center for Biotechnology Information (NCBI), using the *Hc*CCR sequence provided by Leonard et al. (2018) as the query. The resultant alignments allowed us to build the full sequence for the TM regions of *Hc*CCR with high confidence.

Mammalian codon-adapted polynucleotides encoding the TM domains of *Hc*KCR1, *Hc*CCR and *Cr*ChR2 were synthesized and cloned by Genscript Biotech Corporation into the mammalian expression vector pcDNA3.1 (Invitrogen, Cat. #V790-20) in frame with a C-terminal mCherry (*Hc*KCR1 and *Hc*CCR) or EYFP (enhanced yellow fluorescent protein, *Cr*ChR2) tag. The expression construct encoding the TM domain of *Hc*CCR has been deposited in Genbank (accession # OL692497). HEK293 (human embryonic kidney) cells (ATCC, Cat. #CRL-1573; RRID: CVCL_0045) were transfected using the ScreenFect A plus transfection reagent (Fujifilm Waco Chemicals, Cat. #297-77104). All-*trans*-retinal (Sigma-Aldrich, Cat. # R2500) was added from a stock solution in ethanol at the final concentration of 5 μM. Measurements were performed 48–72 h after transfection at room temperature.

Automated whole-cell patch clamp recording was conducted with a SyncroPatch 384i (Nanion Technologies) using planar borosilicate glass medium-resistance chips in a 384-microtiter plate format with one hole per well and Nanion Standard solutions (for their compositions see Supplementary Table 1). Transfected cells were dissociated using TrypLE™ Express, diluted with CHO-S-SFM-II medium (both from ThermoFisher, Cat.# 12604013 and 31033020, respectively) and resuspended in External Physiological solution (Nanion Technologies) at 10^5^-4 × 10^5^ cells ml^–1^. Each well was filled with 30 μl Chip Fill solution, to which 20 μl of the cell suspension was added. Seal formation was enhanced by the addition of 40 μl of the External Physiological solution supplemented with 10 mM CaCl_2_ (final concentration). After formation of GΩ seals, 50 μl of the external solution mixture was replaced with 40 μl of External Physiological solution to reduce the Ca^2+^ concentration. For measurements of the pH dependence, the pH of all solutions except that used for resuspension of the cells was adjusted prior to the experiment to 5.4 or 9.4. The final pH during recording was 5.8 or 8.6, as measured in a solution mixture that mimicked that produced by the SyncroPatch. The internal solution in all experiments was KF 110 Internal (Nanion Technologies). All measurements were carried out at room temperature (25°C). Calculation of the series resistance (R_*s*_) was automatically performed during all recordings, and the recordings with R_*s*_ > 10 MΩ were not included in the analysis.

Photostimulation was provided with LUXEON Z Color Line light-emitting diodes (LEDs) (Lumileds), Cat.# LXZ1-PB01 (470 ± 20 nm, 38 lm at 500 mA, 25°C) or Cat.# LXZ1-PM01 (530 ± 30 nm, 118 lm at 500 mA, 25°C) arranged in a 6 × 16 matrix that covered a quarter of the 384-well chip. The 10–90% rise time for both LED types was < 100 ns. Other technical characteristics of the LEDs, such as spectral power distribution, radiation patterns, color bin structure and mechanical dimensions are provided on the manufacturer’s website.^2^ For green LEDs (LXZ1-PM01), two prototype modifications of the photostimulation hardware were tested. In the first modification (Supplementary Figure 1A), the LED matrix was simply placed on top of the amplifier grid. In the second modification (Supplementary Figure 1B), the matrix was encapsulated in an adaptor that could be fixed on top of the grid in a precise position. Furthermore, a thin class IP68 light guide with a counterbore round head (Mentor, Cat. #1292.1601; length 11.5 mm, diameter 2.2 mm) was attached to each LED to bring the light closer to the cells. In the first modification, the maximal available forward current was 475 mA, in the second, 900 mA. The blue LEDs (LXZ1-PB01) were available only in the first modification of hardware. With both modifications, the matrix had to be repositioned four times during each experiment to cover the entire chip. According to the manufacturer’s data, the dependence of luminosity on the forward LED current was close to linear (Supplementary Figure 1C). The LEDs were driven by a derivative of the CardioExcyte 96 SOL (Nanion, Cat. #191003) and controlled by Biomek commands in combination with a custom stand-alone software provided by Nanion. Variation of the time delay between the programmed and actual light onset is shown in Supplementary Figure 1D.

For data acquisition, PatchControl384 v. 1.9.0 (Nanion Technologies) software was used. The acquisition rate was 200 μs per point (5 kHz sampling rate). The SyncroPatch output was filtered with an analog Bessel filter at 3 kHz and a digital low-pass Lanzcos filter at 3 kHz. The data were analyzed by DataControl384 software v. 1.9.0 (Nanion Technologies). The current traces were also exported in the text format and analyzed by ClampFit, a subroutine of pClamp 10.7 software (Molecular Devices). The kinetics of the current rise and decay was evaluated, respectively, by single and double exponential approximation in ClampFit. Further analysis was performed by Origin Pro 2016 software (OriginLab Corporation). Desensitization was calculated as the difference between the peak and desensitized current divided by the peak current (in %).

Control MPC measurements were performed with an Axopatch 200B amplifier (Molecular Devices) using the same solutions as in the SyncroPatch experiments. The low-pass filter of the amplifier output was set to 2 kHz. The signals were digitized with a Digidata 1440A (Molecular Devices) at 5 kHz sampling rate (200 μs per point) using pClamp 10. Patch pipettes with resistances of 2–4 MΩ were fabricated from borosilicate glass. Continuous light pulses were provided by a Polychrome IV light source (T.I.L.L. Photonics GmbH) in combination with a mechanical shutter (Uniblitz Model LS6, Vincent Associates; half-opening time 0.5 ms). Maximal irradiance at the focal plane of the 40 × objective lens was ∼6.4 mW mm^–2^ at 530 nm and was attenuated using neutral density filters.

Descriptive statistics was calculated by Origin software. The data are presented as mean ± sem values, as indicated in the figure legends; the data from individual replicates are also shown when appropriate. The sample size was estimated from previous experience and published work on similar subjects, as recommended by NIH guidelines. No normal distribution of the data was assumed; when a specific statistics hypothesis was tested, the non-parametric Mann-Whitney test (implemented in Origin software) was used.

## Results

### Seal quality and stability

To test the quality and stability of the seals obtained using the SyncroPatch, we expressed *Hc*KCR1 in HEK293 cells using chemical transfection (for more detail see section “Materials and methods”). The *H. catenoides* genome encodes three ChR paralogs (Leonard et al., 2018). *Hc*KCR1 and *Hc*KCR2 exhibit higher relative permeability for K^+^ over Na^+^, as determined by MPC (Govorunova et al., 2022). To the best of our knowledge, the third paralog had not yet been tested by heterologous expression and patch clamp electrophysiology. The SyncroPatch allows monitoring the resistance at predetermined time points during the entire experiment. Supplementary Figure 2A shows changes in the seal resistance of four representative wells during the execution of the CellCatch command that applies 10-ms voltage steps (from –20 to –30 mV) to test the seal resistance. As evident from the increase in the resistance starting ∼15 s after the addition of the cells, wells 1–3 had successfully captured the cells, whereas well 4 captured no cell. The success rate of the cell capture (>10 MΩ resistance after the addition of the cells to the wells) was ∼85% in our experiments. Supplementary Figure 2B shows the percentage of captured cells that showed a membrane resistance (R_*m*_) > 500 MΩ after the addition of the seal enhancer (10 mM CaCl_2_), wash with the external solution, formation of the whole-cell configuration, and each of the four applications of the voltage step protocol (from –100 to 20 mV in 20-mV steps) in six independent experiments. The seals demonstrated excellent stability during all these manipulations. The dependence of the R_*m*_ on the holding voltage is shown in Supplementary Figure 2C. The duration of a typical experiment was ∼20 min from the time of the cell capture, including the time needed to relocate the LED board.

### Optimization of photostimulation

The tested version of the SyncroPatch does not have integrated photostimulation capacity, so one of the aims of our study was side-by-side comparison of the two prototypes of the add-on photostimulation unit (Supplementary Figures 1A,B; for more detail see section “Materials and methods”). Representative photocurrent traces generated by *Hc*KCR1 in response to light pulses produced by the two modifications of the photostimulation unit are shown in Supplementary Figures 3A, B, top traces. The first version of the photostimulation unit, in which the LED array was directly placed on top of the Faraday cage enclosing the chip, produced rapid (one data point, i.e., < 200 μs) light artifacts of variable amplitude accompanying switching the light on and off (Supplementary Figure 3A, middle and bottom traces), which could be removed digitally (Supplementary Figure 3A, top trace). The second version of the photostimulation unit, with the lightguides attached, produced no such artifacts. Non-transfected cells generated no photoresponses except the abovementioned artifacts with the first version of the photostimulation unit (Supplementary Figures 3A,B, bottom traces). Comparison of the current kinetics clearly showed that the second version produced stronger light, as only in this case the current traces exhibited a peak followed by a decrease to a lower steady-state level (a phenomenon known as “desensitization”). In Supplementary Figures 3C,D we show the current amplitude at the end of a 200-ms light pulse recorded from all 384 individual wells, and Supplementary Figures 3E,F, the corresponding histograms. The cell-to-cell variation of the current amplitude was consistent with variation of the fluorescence and photocurrents measured from similar cultures in MPC experiments. The current amplitude averaged over all 384 wells (including those that captured no cells or non-fluorescent cells) was 28 ± 5 and 81 ± 5 pA with the first and second version, respectively, which confirmed that the second modification of the hardware produced stronger light. Next, we carried out a detailed analysis of the dependence of *Hc*KCR1 photocurrents on the light intensity using the optimized photostimulation unit.

### Light intensity dependence

**FIGURE 1:**
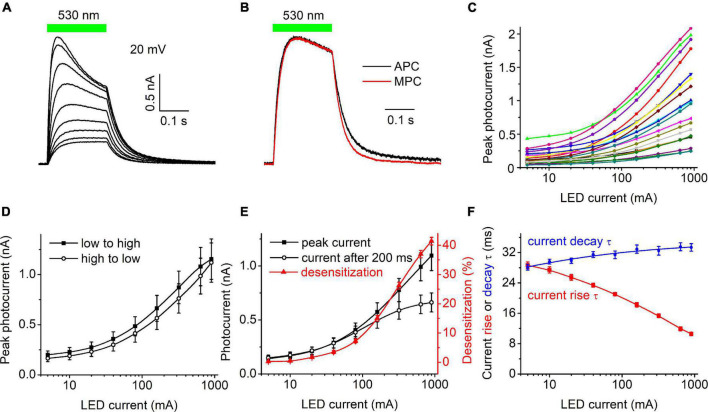
**(A)** A series of photocurrent traces generated by *Hc*KCR1 at 20 mV in response to 200-ms light pulses of incremental intensity. The bar on top shows the duration of illumination. **(B)** Comparison of the normalized photocurrent traces recorded by automated patch clamp (APC, black) at 40 mA forward LED current and by manual patch clamp at 0.2 mW mm^–2^ (MPC, red). **(C)** The dependence of the peak current amplitude on the light intensity (LED current) for 20 individual cells. **(D)** The mean curves for the responses measured upon variation of the LED current from low to high and from high to low values. **(E)** The mean curves for the peak current and desensitized current at the end of the 200-ms light pulse (black symbols and lines, left axis). The red symbols and line (right axis) show the degree of desensitization calculated as the difference between the peak current and the current at the end of 200-ms illumination divided by the peak current and multiplied by 100%. The data points in **(C,E)** are connected with spline lines. **(F)** The dependence of photocurrent rise τ (red) and the main (fast) decay τ (blue) on the light intensity. The data points for the rise τ are connected with a B-spline line; the data for the decay τ are approximated with a logistic function.

Figure 1A shows a representative series of photocurrent traces recorded from a single cell expressing *Hc*KCR1 at 20 mV holding voltage in response to light pulses at incrementally increased LED forward current and thus light intensity. According to the manufacturer’s data, the LED luminosity almost linearly increased upon an increase in the forward current (Supplementary Figure 1C). Comparison of the current kinetics recorded with the SyncroPatch with that recorded at the same voltage and ionic conditions by MPC using a calibrated light source (Figure 1B) showed that the LED output at 40 mA forward current roughly corresponded to 0.2 mW mm^–2^. Figure 1C shows the dependence of the peak current on the LED forward current for 20 individual cells that generated the largest response. The mean dependence measured when the intensity was increased from low to high closely matched that measured when the intensity was changed in the reversed order (Figure 1D), which indicated that the 30-s interval between subsequent light pulses was sufficient for the full recovery of *Hc*KCR1, and that only minimal bleaching/rundown was observed. The amplitude of the photocurrent measured at the end of 200-ms illumination saturated earlier than the peak (Figure 1E), as in other ChRs studied earlier by MPC (Ishizuka et al., 2006; Ernst et al., 2008). The dependence of desensitization on the LED forward current is shown as the red symbols and line in Figure 1E. Figure 1F shows the dependence of the time constants (τ) of the photocurrent rise (red) and decay (blue) on the LED current.

### Current-voltage relationships of three channelrhodopsin variants and their dependence on external pH

**FIGURE 2:**
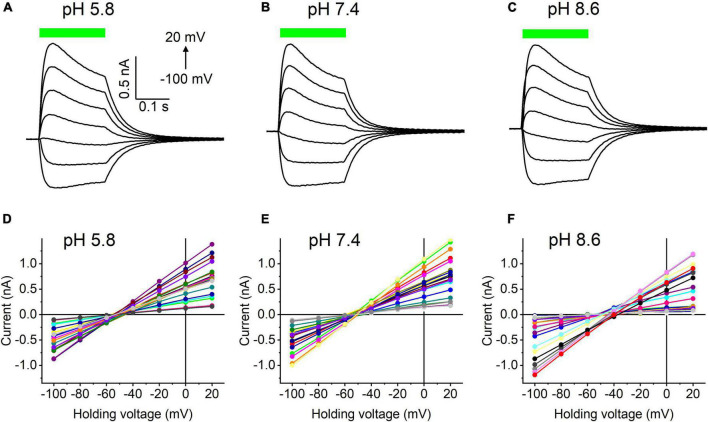
**(A–C)** Photocurrent traces recorded from *Hc*KCR1 at the indicated external pH in response to a 200-ms light pulse (530 nm, 900 mA), the duration of which is shown as a colored bar on top. The holding voltage was varied from –100 to 20 mV in 20-mV steps. **(D–F)** Current-voltage relationships of *Hc*KCR1 measured in individual cells. The current amplitude was measured at the end of a 200-ms light pulse.

**FIGURE 3:**
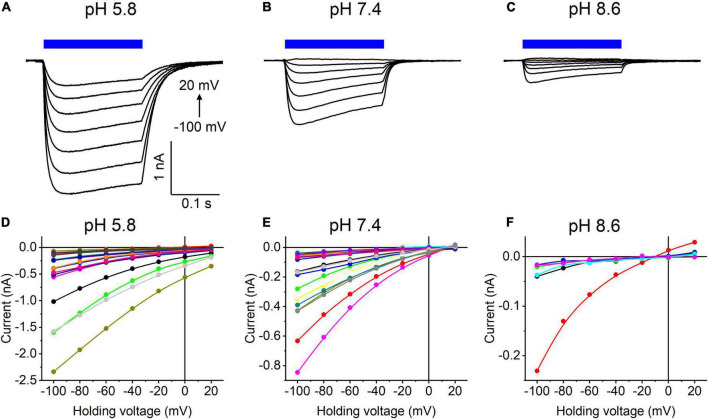
**(A–C)** Photocurrent traces recorded from *Cr*ChR2 at the indicated external pH in response to a 200-ms light pulse (470 nm, 475 mA), the duration of which is shown as a colored bar on top. The holding voltage was varied from –100 to 20 mV in 20-mV steps. **(D–F)** Current-voltage relationships of *Cr*ChR2 measured in individual cells. The current amplitude was measured at the end of a 200-ms light pulse.

**FIGURE 4:**
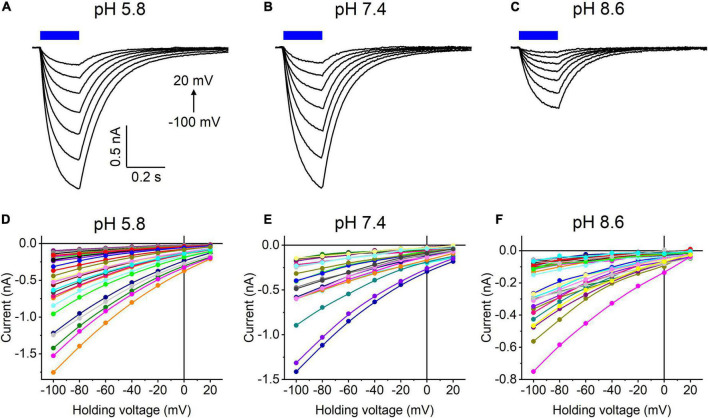
**(A–C)** Photocurrent traces recorded from *Hc*CCR at the indicated external pH in response to a 200-ms light pulse (470 nm, 475 mA), the duration of which is shown as a colored bar on top. The holding voltage was varied from –100 to 20 mV in 20-mV steps. **(D–F)** Current-voltage relationships of *Hc*CCR measured in individual cells. The current amplitude was measured at the end of a 200-ms light pulse.

Our previous MPC study showed that *Hc*KCR1 exhibits > 100 times less permeability for protons than *Cr*ChR2 (Govorunova et al., 2022). This is very unusual among known CCRs, so we sought to verify this result with the SyncroPatch. Representative series of photocurrent traces generated by *Hc*KCR1 at incrementally increased holding voltages at pH 5.8, 7.4, and 8.6 are shown in Figures 2A–C. Our analysis of *Hc*KCR1 photocurrents by MPC showed that its P_*K*_/P_*Na*_ permeability ratio decreases during continuous illumination (Govorunova et al., 2022). This decrease explains the biphasic (first positive, then negative) photocurrent trace recorded at –60 mV in Figure 2A. The current-voltage relationships (*IE* curves) for individual cells that generated the largest response are shown in Figures 2D–F. We used well-characterized *Cr*ChR2 as a positive control for proton permeability. Representative series of photocurrent traces generated by *Cr*ChR2 under the same ionic conditions as used for *Hc*KCR1 are shown in Figures 3A–C, and the corresponding *IE* curves, in Figures 3D–F. In contrast to *Hc*KCR1, alkalization strongly suppressed *Cr*ChR2 photocurrents (in fact, at pH 8.6 only a few cells generated currents that could be resolved from the noise). Figure 4 shows the results of characterization of the third ChR paralog from *H. catenoides* using the SyncroPatch. Despite its 74.4% primary structure identity and 87% similarity with *Hc*KCR1 (Supplementary Figure 4), the *E*_*rev*_ of photocurrents generated by this ChR was positive under our conditions (Figure 4), which indicated that it was more permeable for Na^+^ than K^+^. Therefore, we named this protein *Hc*CCR for “*H. catenoides* cation channelrhodopsin.”

**FIGURE 5:**
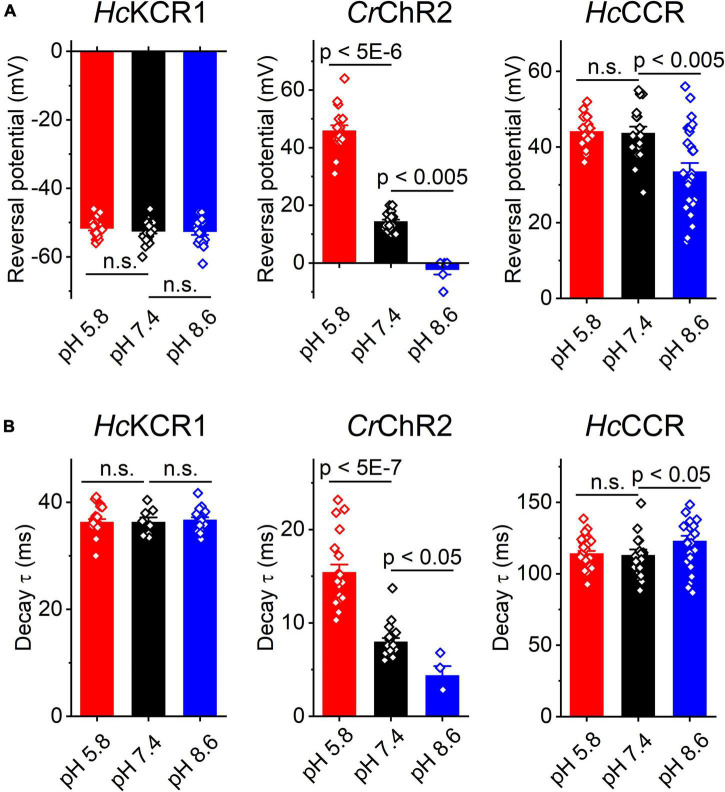
The reversal potential of photocurrents **(A)** and the time constant (τ) of the main (fast) component of photocurrent decay **(B)** in *Hc*KCR1, *Cr*ChR2 and *Hc*CCR measured at the indicated external pH. The bars are mean ± sem, the circles are the data from individual cells. Numerical data and full statistical analysis are provided in Supplementary Datasheet 2.

Comparison of the *E*_*rev*_ values for all three tested ChRs are shown in Figure 5A. The *E*_*rev*_ of *Hc*KCR1 photocurrents was strongly negative and showed no dependence on pH in the tested range, which was consistent with the results of our previous MPC study (Govorunova et al., 2022). In contrast, the *E*_*rev*_ of *Cr*ChR2 shifted upon variation of pH, which matched previous observations by MPC (Nagel et al., 2003; Tsunoda and Hegemann, 2009). The *E*_*rev*_ of *Hc*CCR at pH 7.4 was more positive than that of *Cr*ChR2, indicating that *Hc*CCR exhibits a higher Na^+^/K^+^ permeability ratio than *Cr*ChR2. The *E*_*rev*_ of *Hc*CCR exhibited only a small *E*_*rev*_ shift upon alkalization, indicating its lower relative permeability for protons than that of *Cr*ChR2.

As shown previously by MPC, the decay kinetics of *Cr*ChR2 photocurrents accelerates upon alkalization (Tsunoda and Hegemann, 2009). The SyncroPatch (Figure 5B) recordings reproduced this effect. In contrast to *Cr*ChR2, the decay of *Hc*KCR1 photocurrents was independent of pH, whereas the decay of *Hc*CCR photocurrents slightly slowed at alkaline pH.

## Discussion

As of this writing, APC platforms have been used mostly for characterization of mammalian voltage- and ligand-gated ion channels and drug discovery (Brinkwirth et al., 2020; Potet et al., 2020; Bell and Fermini, 2021; Obergrussberger et al., 2021, 2022). Here we tested the SyncroPatch 384i for characterization of ChRs, light-gated ion channels from eukaryotic microbes, expressed in model human cells as C-terminal EYFP or mCherry fusions. Planar APC using the SyncroPatch offers an important advantage over MPC or even pipette-based APC (Kolb et al., 2019), as it completely eliminates the bias introduced by the investigator’s selection of fluorescent cells for patching. However, this advantage is relevant only if all cells express the transgene. Stably transfected cell lines have been created for *Cr*ChR2 (Zimmermann et al., 2006), but not for *Hc*KCR1 and *Hc*CCR. Therefore, we used transient chemical transfection to avoid time-consuming generation of stable lines, as we had access to the SyncroPatch 384i for only a limited time. The efficiency of chemical transfection in our experiments, estimated by microscopic observation of tag fluorescence, was 50-70%, which explained the absence of photocurrents in some cells captured in a multiwell plate. Therefore, using chemical transfection saved time, but reduced the throughput. Viral delivery of transgenes yielding higher transfection efficiency may provide a reasonable compromise between time and throughput.

Variability of the time delay between the programmed and actual onset of illumination (Supplementary Figure 1D) needs to be taken into account to avoid introducing errors during automatic measurements of photocurrent amplitudes by DataControl384 software. This variability can easily be accounted for if the recorded traces are exported and analyzed individually by ClampFit or other relevant software. According to Nanion engineers, this variability is due to the Biomek command used to trigger the LEDs, not to the LEDs themselves. The SyncroPatch 384i platform that we used was based on the Biomek i5 robot, recently replaced with the Biomek 4000 robot. It remains to be seen whether this variability is eliminated in the new SyncroPatch 384 model based on the upgraded robot. We found that the improved photostimulation unit for the SyncroPatch (Supplementary Figure 1B) that was available only for 530-nm LEDs produced sufficiently intense light to cause desensitization (attenuation of photocurrent during continuous illumination) of the tested ChRs. Desensitization is observed in all known ChRs and needs to be minimized for optogenetic purposes. Desensitization is caused by accumulation of long-lived non- or less-conductive intermediate(s) of the photocycle, but its molecular mechanisms are poorly understood and appear to be different in different ChRs (Saita et al., 2018; Kuhne et al., 2019; Oppermann et al., 2019; Sineshchekov et al., 2020). The SyncroPatch is expected to be invaluable for high-throughput screening of random ChR mutants for decreased desensitization.

Measurements of the *IE* curves require high seal quality and stability to avoid “seal leakage currents” that develop in response to applied voltage gradients (Wilson et al., 2011). The SyncroPatch provides an excellent seal stability upon application of repetitive voltage steps (Supplementary Figure 2). Our results obtained with *Hc*KCR1 and *Cr*ChR2 using this instrument are consistent with those of previous MPC studies (Nagel et al., 2003; Govorunova et al., 2022), which validates using the SyncroPatch for measurements of the *IE* curves and determination of the ionic selectivity of ChRs. In our analysis of the pH dependence of ChR photocurrents and current-voltage relationships, we used external solutions with preadjusted pH. The SyncroPatch also allows rapid exchange of the external and internal solutions without disturbing the gigaohm seals, as shown in experiments with voltage- and ligand-gated channels (Toh et al., 2020; Rotordam et al., 2021; Obergrussberger et al., 2022).

Using the SyncroPatch we found that the third ChR from *H. catenoides*, named here *Hc*CCR, showed no preferred selectivity for K^+^ over Na^+^, in contrast to its close relatives *Hc*KCR1 and *Hc*KCR2 from the same organism. In fact, the Na^+^/K^+^ permeability ratio of *Hc*CCR is even higher than that of *Cr*ChR2, as is evident from the comparison of the corresponding *E*_*rev*_ values at pH 7.4 (Figure 5A). The close sequence homology of *Hc*KCR1 and *Hc*CCR (Supplementary Figure 4) will potentially benefit identification of residue motifs responsible for the high K^+^/Na^+^ permeability ratio of *Hc*KCR1.

Our conclusion is that the SyncroPatch 384i from Nanion accelerates determination of the basic biophysical characteristics of ChRs, such as their light intensity dependence and current-voltage relationships, and facilitates screening of ChR homologs identified in global polynucleotide sequencing projects. An upgrade of the photostimulation hardware for simultaneous illumination of the entire 384-well chip is expected to further increase its efficiency.

## Data availability statement

The datasets presented in this study can be found in online repositories. The names of the repository/repositories and accession number(s) can be found in the article/Supplementary material.

## Author contributions

EG, OS, and JS conceived the study. LB identified and refined the *Hc*KCR1 and *Hc*CCR sequences in the public databases. EG carried out APC and MPC experiments, analyzed their results, prepared the figures and wrote an original draft. All authors contributed to its review and editing.

## Footnotes

1
https://www.ebi.ac.uk/biostudies/studies/S-BSST46

2
https://lumileds.com/wp-content/uploads/files/DS105-LUXEON-Z-Color-Line-datasheet.pdf
