# Supplementary material for: Biophysical characterization of light-gated ion channels using planar automated patch clamp
Source: Front Mol Neurosci. 2022 Aug 9;15:976910. doi: 10.3389/fnmol.2022.976910 (PMC9396214; doi:10.3389/fnmol.2022.976910)
Supplement: Supplementary file 1 [file Data_Sheet_1.docx]

Supplementary Material

# Supplementary Figures and Tables

##
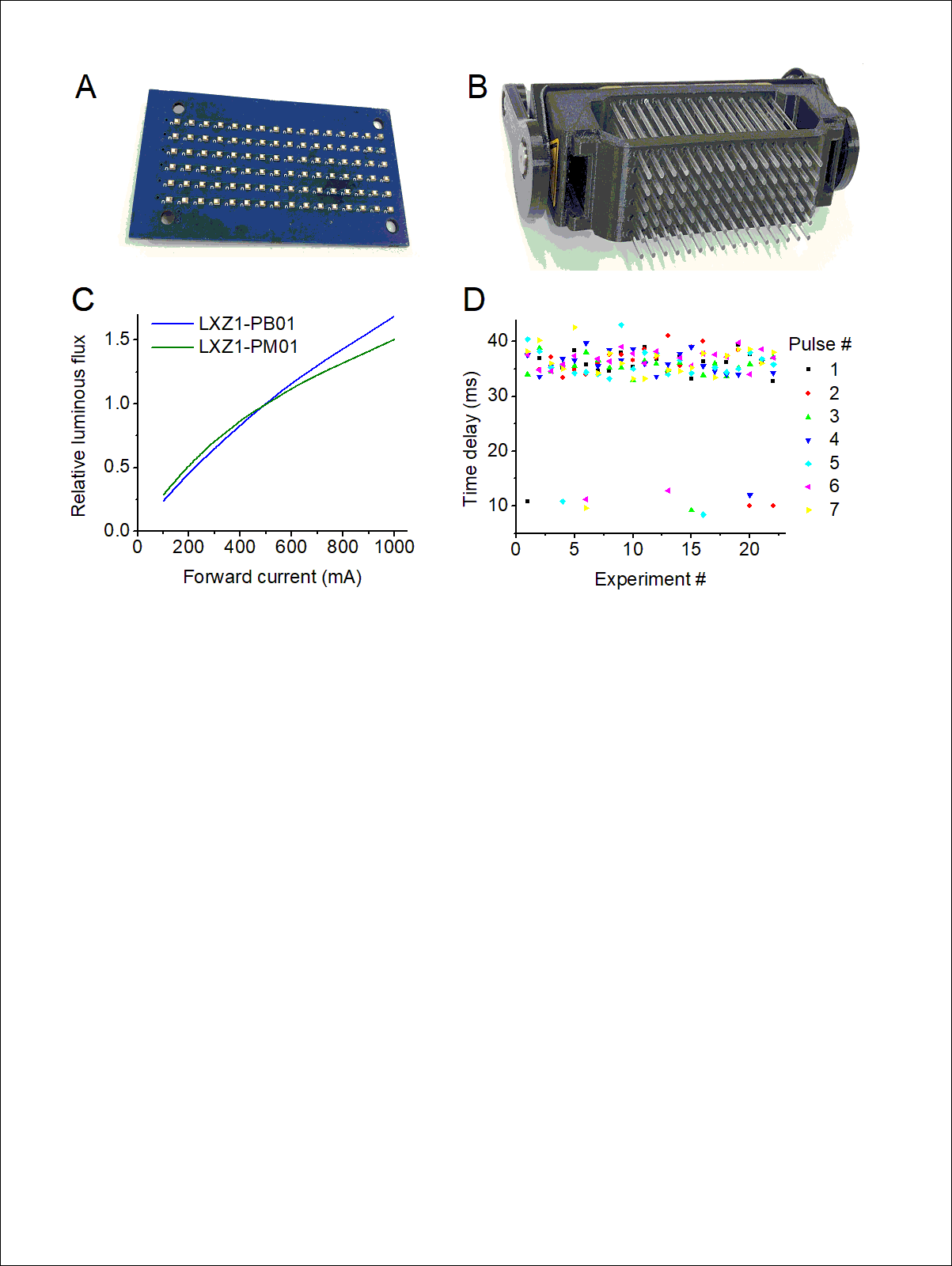
Supplementary Figures

**Supplementary Figure 1.** (A and B) Two modifications of the photostimulation hardware used in our experiments. The first version (A) consists of a bare LED array placed on top of the Faraday cage containing the chip and the amplifiers. In the second version (B), thin lightguides are attached to individual LEDs to focus the light and bring it closer to the cells. (C) The dependence of relative luminous flux on LED forward current provided by the manufacturer (<https://lumileds.com/wp-content/uploads/files/DS105-LUXEON-Z-Color-Line-datasheet.pdf>). (D) The delay between the programmed and actual onset of illumination in our experiments using LXZ1-PM01 LEDs. In each of 22 individual experiments, seven successive 200-ms light pulses were programmed with the time interval 30 s. The data points are the values measured for individual pulses. Most pulses (144 out of 154 total) were delivered with a fixed delay 36 ± 0.2 ms (mean ± sem), but 10 pulses were delivered prematurely.

**Supplementary Figure 2.** (A) The time course of changes in the seal resistance during the cell capture. The recordings from four representative wells are shown. Wells 1-3 captured the cell, whereas well 4 did not. The recording started when the tips move into the wells. The cells were dispensed during the time from ~6 s to ~16 s. (B) The percentage of the captured cells with the membrane resistance (R_m_) >500 MΩ measured at the key time points in six independent SyncroPatch experiments shown by different symbol shapes. pH of the external solution is indicated by colors explained in the figure legend. SE, after the addition of the seal enhancer; Wash, after washing the cells with the external solution; V1-V4, after application of four voltage step protocols each consisting of seven 20-mV steps (from -100 to 20 mV). (C) Changes in the R_m_ during execution of the voltage protocol in a typical experiment. The data points are mean ± sem (n = 217 cells with R_m_ >500 MΩ at the beginning of the measurements).


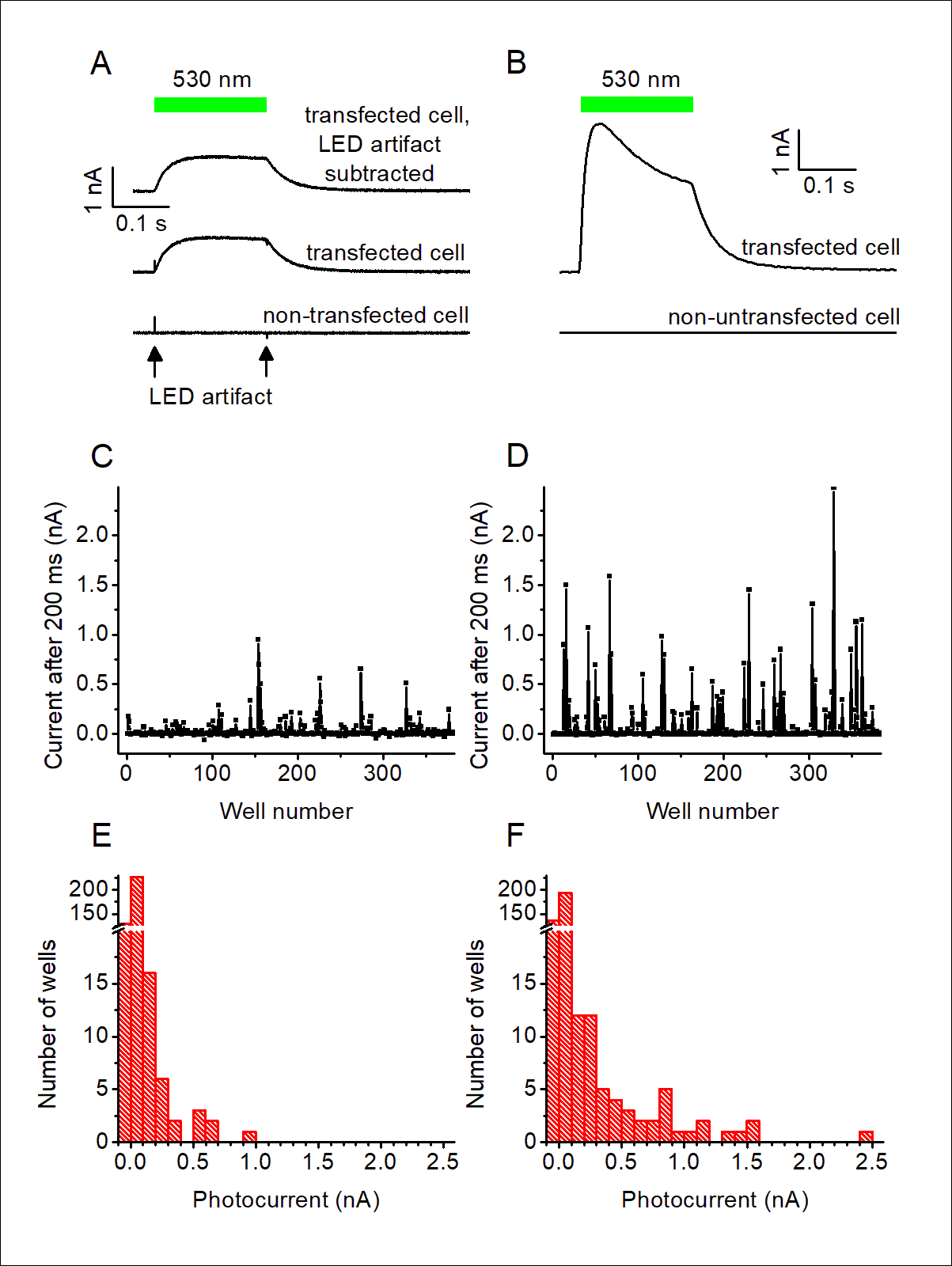


**Supplementary Figure 3.** (A and B) Current traces recorded from cells transfected with *Hc*KCR1 (top traces) and from non-transfected cells (bottom traces) in response to 200-ms illumination with two modifications of the photostimulation hardware shown in Supplementary Fig. 1A and B, respectively. The arrows in A point to the rapid LED artifact associated with switching the LEDs on and off. The middle trace in A shows the same trace as at the top prior to the digital removal of the LED artifacts. The green bars show the duration of illumination. Note the different kinetics of photocurrents, indicating that second modification of the hardware produced stronger light. (C and D) The photocurrent amplitude measured at the end of a 200-ms light pulse in each individual well using the two photostimulation hardware modifications. The wells showing no current contained no cells or non-transfected cells. (E and F) Histograms of the data from panels C and D, respectively.

***|* *|* *|* *|*
*HcK*CR1 MPFYDSRPPEGWPKGSINDMDYPLLGSICAVCCVFVAGSGIWMLYRLDLG 50
*HcC*CR MPFCGGRPEDGWHHGSIHDMDYPLLGAMAAICSVFIGGSGAWMLYRLDLG 50
 *|* *|* *|* *|*
*HcK*CR1 MGYSCKPYKSGRAPEVNSLSGIICLLCGTMYAAKSFDFFDGGGTPFSLNW 100
*HcC*CR LGYSCKPHHSGYAPEANSFSALSCLVSGTIYAAKTFDFFDGGGTPFSFNW 100
 *|* *|* *|* *|*
*HcK*CR1 YWYLDYVFTCPLLILDFAFTLDLPHKIRYFFAVFLTLWCGVAAFVTPSAY 150
*HcC*CR YWYLDYVFTCPLILLDVLYTLEIPHKLRFVFAVIITLWCGVAAFVTPSAF 150
 *|* *|* *|* *|*
*HcK*CR1 RFAYYALGCCWFTPFALSLMRHVKERYLVYPPKCQRWLFWACVIFFGFWP 200
*HcC*CR RFGYYAVGCVWFVPFSFSLLRHVKQRYQVYPPKCQKLLFWACTIFFGFWP 200
 *|* *|* *|* *|*
*HcK*CR1 MFPILFIFSWLGTGHISQQAFYIIHAFLDLTCKSIFGILMTVFRLELEEH 250
*HcC*CR LFPILFLFSWLGTGHIDQQAFTIIHAFLDLFCKTVFGLIMTFFRLELEEH 250
 *|*
*HcK*CR1 TEVQGLPLNEPETLS 265
*HcC*CR TEVLGLPLNEPKGKH 265**

**Supplementary Figure 4.** The amino acid alignment of the rhodopsin domains of *Hc*KCR1 (Genbank accession # MZ826861) and *Hc*CCR (Genbank accession # OL692497). The full lengths of the polypeptides encoded by the corresponding genes are 395 and 432 residues, respectively, but the remaining C-terminal residues are not required for the channel function. The identical residues are shaded black. The numbers are the last residue numbers in each line. The ticks mark every 10^th^ residue.

## Supplementary Tables

**Supplementary Table 1.** Solution compositions in SyncroPatch experiments.

Abbreviations: EGTA, ethylene glycol tetraacetic acid; HEPES, 4-(2-hydroxyethyl)-1-piperazineethanesulfonic acid. All concentrations are in mM.

|  | **NaCl** | **KCl** | **KF** | **CaCl_2_** | **MgCl_2_** | **EGTA** | **HEPES** | **Glucose** | **pH** |
| --- | --- | --- | --- | --- | --- | --- | --- | --- | --- |
| **Nanion Internal KF** | 10 | 10 | 110 | — | — | 10 | 10 | — | 7.2 |
| **Nanion External Chip Fill** | 140 | 4 |  | — | — | — | 10 | 5 | 7.4 |
| **Nanion External Physiological** | 140 | 4 |  | 2 | 1 | — | 10 | 5 | 7.4 |
| **Nanion Seal Enhancer Physiological** | 140 | 4 |  | 10 | 1 | — | 10 | 5 | 7.4 |
